# Humanitarianism

**Published:** 1910-12-24

**Authors:** 


### The Hospital

A JOURNAL OF

The Medical Sciences and Hospital Administration.

NEW SERIES. NO. 203, VOL. VIII. [NO. 1275, VOL. XLIX.] SATURDAY, DECEMBER 24 1910.

### HUMANITARIANISM.

THE asperities of party politics having been happily concluded for the time being, the mere reaction after a year of so much strife should produce more than ordinary good-will among mankind this Christmas. Humanity, charity, sympathy, in the full senses of those often misused terms, are gradually perfusing themselves throughout civilisation, and at this season especially it is possible to observe the growth of kindly feeling between man and man, and even between nation and nation. Steadily but slowly the world is being uplifted nearer to ideals which it will take many centuries yet to approach. Human nature changes little through the ages; indeed, cynics would have us believe that it does not change at all. This assertion is in flat contradiction of all the teachings of evolution, though it may be admitted that the alteration is extremely slow compared with the enormous change in man's environment during the last thirty or forty centuries. That tardiness must be recognised and remembered whenever changes, having important bearings on that organisation for mutual protection which is called society, are under discussion.

The outbreak of misplaced and visionary energy which prompted Mr. A. C. Benson to write to the *Times* some three weeks ago a letter advocating the abolition of capital punishment shows forgetfulness of these principles. Let it be noted in passing that the agitation for the abolition of this institution is conducted from two entirely distinct standpoints. One, which may be called the ultra-humanitarian view, is that capital punishment for murder is brutal and shocking to the susceptibilities of certain people (the humanitarians), as well as very unpleasant for the murderer. The other is that of certain criminologists, who argue that it does not even achieve the avowed end and aim—that is, deterrence. The latter objection is a matter of opinion upon which criminologists are entitled to speak, provided they are really in touch with those lowest strata of society wherein crimes of violence are commonest and temptations to commit them greatest—not merely arm-chair philosophers swayed by the theorisings of Continental *savants* from countries where human life and human freedom are valued less highly than they have always been in Britain. The letter to which we have already referred has at least been useful in eliciting very emphatic remarks which contradict this opinion of those whom, without offence, one may call the *dilettanti* criminologists. Several of those who are best qualified to know what the most degenerate sections of our populace feel, testify that there is a most wholesome terror among them of the last penalty of the law; not merely of all capital punishment in the abstract, but especially of that form of it which prevails in these islands. Thus Mr. Benson's letter has served to draw forth comments which are both unfavourable to his own opinions and also destructive of the arguments of those others who, on different grounds, desire with him the abolition of capital punishment.

Into the details of Mr. Benson's theme there is no need to follow him here, particularly as the subsequent correspondence—one conducted on a level as high as any which has adorned the great newspaper in which it was published—must be fresh in the minds of all who have read it. But as a sign of the times the incident has a moral which medical men have a right to note, for it is one with which they are already becoming familiar. Call it psychasthenia, hysteria, or any other name that is the vogue of the moment, there is perceptible a want of fibre, a lack of hardiness and manliness, in the character of some of those who have the public ear which is but the reflection of much that the profession encounters on its daily rounds. It would take a Carlyle to denounce this peculiar spirit, or lack of spirit, in terms which would arouse the nation to a perception of this weakness; but medical men can do something to encourage greater decision, manliness, and moral sturdiness among the population at large.

The letters of Sir Leslie Stephen and others in reply to Mr. A. C. Benson show clearly that those to whom the thought of the condemned man in his cell or on the scaffold is akin to mental anguish are quite neglectful of the agonies which the wanton murderer callously inflicts on his victim and on those to whom that victim is dear. The fiendish crimes carried out in perfectly cold blood by Neil Cream, Chapman, Charles Peace, and recently by Crippen, may conceivably be evidence of some mental abnormality in their perpetrators; but, if so, it is clear enough that to abolish capital punishment is to invite an epidemic of the disorder. To adopt the alternative suggested in Mr. Benson's euthanasian creed is likely to be equally ineffectual prophylaxis. When potential murderers can reflect that there is a very fair chance of their escaping punishment of death entirely, and that the worst they have in prospect is a lethal chamber postponed to suit their convenience, there is a fair chance that the potentiality may materialise in cases where it otherwise would not. Even to abolish the death sentence for those acting in hot blood under great provocation would be a dangerous experiment, and one which the facts and figures of foreign countries should render us very indisposed to try. When the most brutal and calculating criminals are those on whose behalf petitions are most freely signed, as is becoming the established custom, and when, as in the case of the murder of Mr. Whiteley, parricide is regarded by the populace as a palliation rather than an aggravation of an ordinary murder, one may well wonder where this false and morbid sentimentality will cease.

